# Jaw Periosteum-Derived Mesenchymal Stem Cells Regulate THP-1-Derived Macrophage Polarization

**DOI:** 10.3390/ijms22094310

**Published:** 2021-04-21

**Authors:** Fang He, Felix Umrath, Siegmar Reinert, Dorothea Alexander

**Affiliations:** Department of Oral and Maxillofacial Surgery, University Hospital Tübingen, 72076 Tübingen, Germany; fang.he@med.uni-tuebingen.de (F.H.); felix.umrath@med.uni-tuebingen.de (F.U.); siegmar.reinert@med.uni-tuebingen.de (S.R.)

**Keywords:** jaw periosteal cells, immunomodulation, macrophage polarization, fetal bovine serum, human platelet lysate, coculture

## Abstract

Mesenchymal stem cells from bone marrow have powerful immunomodulatory capabilities. The interactions between jaw periosteal cells (JPCs) and macrophages are not only relevant for the application of JPCs in regenerative medicine, but this understanding could also help treating diseases like osteonecrosis of the jaw. In previous studies, we analyzed, for the first time, immunomodulatory features of 2D- and 3D-cultured JPCs. In the present work, the effects of JPCs on the polarization state of macrophages in contact coculture were analyzed. To improve the macrophage polarization study, different concentrations of PMA (5 nM, 25 nM, and 150 nM) or different medium supplementations (10% FBS, 10% hPL and 5% hPL) were compared. Further, in order to analyze the effects of JPCs on macrophage polarization, JPCs and PMA-stimulated THP-1 cells were cocultured under LPS/IFN-γ or IL-4/IL-13 stimulatory conditions. Surface marker expression of M1 and M2 macrophages were analyzed under the different culture supplementations in order to investigate the immunomodulatory properties of JPCs. Our results showed that 5 nM PMA can conduct an effective macrophage polarization. The analyses of morphological parameters and surface marker expression showed more distinct M1/M2 phenotypes over FBS supplementation when using 5% hPL during macrophage polarization. In the coculture, immunomodulatory properties of JPCs improved significantly under 5% hPL supplementation compared to other supplementations. We concluded that, under the culture condition with 5% hPL, JPCs were able to effectively induce THP-1-derived macrophage polarization.

## 1. Introduction

For a successful application of tissue engineering products, including the implantation of biological scaffolds into recipients, an activation of host immune responses should be avoided in order to prevent implant rejection [1]. The immunomodulatory functions of mesenchymal stem cells (MSCs), including the effective suppression of both innate and adaptive immunity [2,3], could be used to improve implant survival in the body. In response to changes of the microenvironment, MSCs produce numerous chemokines, cell mobilization, and growth factors and regulate the activity of immune cells to maintain the immune homeostasis [4]. For bone regeneration in oral and maxillofacial surgery, jaw periosteal cells (JPCs) are a very promising stem cell source due to their high osteogenic potential and good accessibility. Recently, we described for the first time that JPCs possess similarities to bone marrow MSCs concerning their immunoregulatory functions [5,6]. In cocultures with 2D- and 3D-cultured JPCs, dendritic cell maturation could be effectively attenuated. In the present study, we analyzed the effects of JPCs on macrophage polarization.

Macrophages are multitargeting immune cells that possess phagocytic functions for the digestion of microbial pathogens, foreign substances, and cell debris [7]. Immature macrophages can be polarized into different subtypes of mature macrophages, depending on their microenvironments. Most commonly, classical activation of proinflammatory M1 macrophages is induced by lipopolysaccharide (LPS) and interferon gamma (IFN-γ), while anti-inflammatory M2 (alternative activation) macrophages are generated by interleukin-4 (IL-4) and interleukin-13 (IL-13) stimulation [8]. The recently discovered CD169^+^ and T-cell receptor-positive (TCR^+^) macrophages reflect the polydirectional plasticity of macrophages, depending on multiple microenvironmental signals [9].

The general use of FBS as a growth factor supplement for in vitro cell culture increases the contamination risk with host viruses or bacteria and may induce immune reactions when cultured cells are used for regenerative purposes. For instance, the development of diffuse urticaria has been reported in individuals who received MSCs produced under FBS supplementation [10,11]. The supplementation with human platelet lysate (hPL) instead of fetal bovine serum (FBS) for in vitro cell culture was proposed to be in accordance with the good manufacturing practice (GMP) guidelines. It can be easily separated after apheresis and filtering procedures for research and clinical use [12,13,14]. Compared with FBS, hPL contains a series of potent bioactive mediators primarily stored in α-granules and released after lysation procedure. These factors have many advantages, as already demonstrated in studies analyzing immune and stem cells [15]. Besides promoting cell proliferation and maintaining the stem cell plasticity, hPL seems to enhance further the immunomodulatory properties of MSCs [16]. These findings are partially consistent with our previous research, showing that hPL can effectively increase the proliferation activity and the osteogenic differentiation potential of JPCs [17].

In previous studies on macrophage polarization in vitro, cell cultures were supplemented with FBS [18]. However, in order to study macrophage polarization in vitro, it is particularly important to choose the appropriate stimulation in order to get closer to the in vivo conditions. Using hPL for immunological research has not only the potential to exclude the influence of animal-derived factors but also to simulate the microenvironment within the human body more effectively [19]. Additionally, the comparison of the macrophages’ polarization state under FBS and/or hPL supplementation has not been reported until the present study.

In our study, we analyzed the influence of JPCs on THP-1-derived macrophage polarization in the direct coculture and compared JPCs’ regulatory functions under FBS and/or hPL supplementation.

## 2. Results

### 2.1. Establishment of the Suitable Protocol for Macrophage Polarization

THP-1 cells were induced with 5, 25, or 150 nM PMA for 48 h to differentiate into M0 macrophages. Thereafter, M0 macrophages were further differentiated into M1 or M2 macrophages by addition of LPS (15 ng/mL)/IFN-γ (20 ng/mL) or IL-4 (20 ng/mL)/IL-13 (20 ng/mL) for 24 or 72 h.

**Figure 1:**
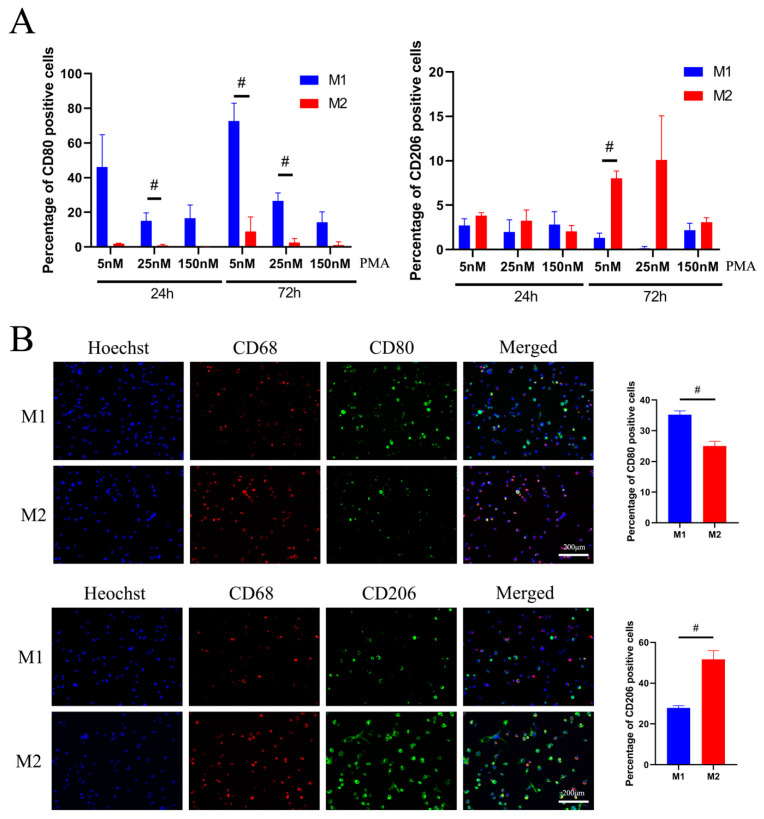
Optimization of the differentiation protocol for M1/M2 polarization by analyzing cell surface markers. (**A**) Flow cytometric measurements of CD80 and CD206 expression under different PMA concentrations for 48 h, and further M1/M2 conditions for 24 or 72 h (*n* = 3, # *p* < 0.05). (**B**) Immunofluorescence staining of M1 and M2 macrophages for CD68, CD80, and CD206 expression counterstained with Hoechst 33342. Representative images and semiquantitative results of CD80 and CD206 expression in M1 or M2 macrophages are shown (*n* = 10 images per group, # *p* < 0.05); scale bar: 200 μm.

When PMA concentrations of 5 or 25 nM were followed by 72 h for M1/M2-polarization, CD80 expression by M1 versus M2 macrophages was significantly increased compared to the results obtained after 24 h (5 nM PMA 72 h CD80: M1 72.69 ± 10.27 versus M2 8.840 ± 4.912, *p* < 0.05; 25 nM PMA 72 h CD80: M1 26.71 ± 4.466 versus M2 2.563 ± 1.383, *p* < 0.05). Under the longer incubation conditions, the expression of CD206 reached significantly higher levels in M2 compared to M1 macrophages (5 nM PMA 72 h CD206: M1 1.310 ± 0.5498 versus M2 8.010 ± 0.8504, *p* < 0.05) (Figure 1A).

Figure 1B illustrates immunofluorescent detection of CD68, CD80, and CD206 expression on M1/M2 macrophages, counterstained with Hoechst 33342 for nuclear staining. The images show that macrophage-specific markers CD68, CD80, and CD206 could be found in both M1 and M2 macrophages. Of M1 macrophages, 32.03% expressed CD68, and 54.37% of M2 macrophages expressed CD68. Additionally, 35.23% of M1 macrophages expressed CD80, and 51.66% of M2 macrophages expressed CD206 on their surface. Semiquantitative results showed significant differences. Compared to M2 macrophages, 10% higher M1 macrophage numbers were CD80-positive. Compared to M1 macrophages, 24% higher M2 macrophage numbers were CD206-positive (Figure 1B).

### 2.2. FBS or hPL Culture Supplementation Induces Different Cellular Morphologies of M1 and M2 Macrophages

**Figure 2:**
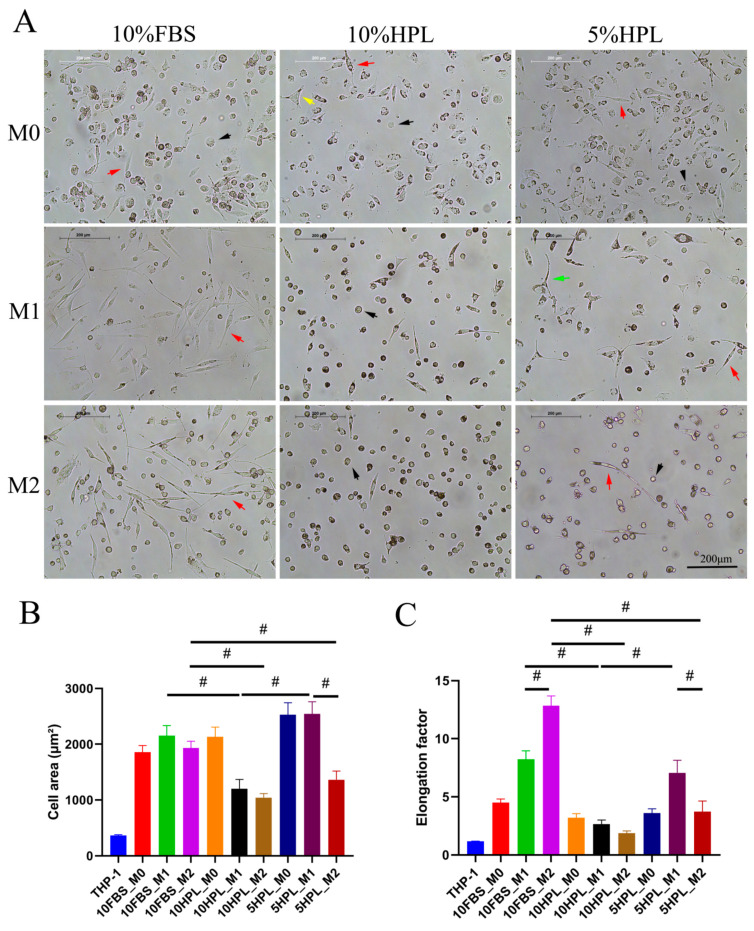
Cell morphology of PMA-(M0)-induced THP-1 cells, which were further differentiated into M1 and M2 macrophages cultured under FBS or hPL supplementation. (**A**) Phase contrast microscopic pictures of M0, M1, or M2 macrophages under 10% FBS, 10% hPL, or 5% hPL culture supplementation (black arrow: round cell; red arrow: fibroblast-like cell; yellow arrow: triangular cell; green arrow: polygonal cell); scale bar: 200 μm. (**B**) Analysis of the cell area of M0, M1, and M2 macrophages (*n* = 30 cells per group, # *p* < 0.05). (**C**) Analysis of the elongation factor of M0, M1, and M2 macrophages (*n* = 30 cells per group, # *p* < 0.05).

Phase contrast microscopic images showed that there were significant morphological differences in induced macrophages’ phenotype under FBS or hPL culture supplementation. THP-1 cells differentiated to M0 macrophages in 10% FBS, while 10% hPL and 5% hPL medium showed round, fibroblast-like, and triangular morphology. Most of the M1 macrophages supplemented with 10% FBS presented a fibroblast-like morphology. Most of the M1 macrophages treated with 10% hPL presented a round morphology. M1 macrophages cultured in 5% hPL displayed polygonal morphology as well as fibroblast-like morphology. Most of the M2 macrophages treated with 10% FBS exhibited a fibroblast-like morphology. M2 macrophages cultured under 10% hPL and 5% hPL supplementation were mostly round with lower numbers of fibroblast-shaped cells (Figure 2A). Quantification of cell area showed no significant difference between M1 and M2 macrophages induced under 10% FBS or 10% hPL supplementation. In contrast, the cell area of M1 macrophages supplemented with 5% hPL was found to be significantly larger than that of M2-type macrophages (5% hPL: M1 2543 ± 217.0 versus M2 1363 ± 155.0, *p* < 0.05). Compared to M2 macrophages cultured under 10% FBS conditions, the area of M2 macrophages supplemented with 5% hPL was significantly lower (M2: 10% FBS 1935 ± 119.4 versus 5% hPL 1363 ± 155.0, *p* < 0.05) (Figure 2B). Analysis of the elongation factor showed that M1 and M2 macrophages exhibited opposite patterns when cultured under 10% FBS or 5% hPL supplementation. Using 10% FBS, the elongation factor of M1 macrophages was significantly lower than that of M2 macrophages (10% FBS: M1 8.227 ± 0.7317 versus M2 12.84 ± 0.8333, *p* < 0.05). Using 5% hPL, the elongation factor of M2 macrophages was significantly lower than that of M1 macrophages (5% hPL: M1 7.070 ± 1.068 versus M2 3.728 ± 0.9190, *p* < 0.05). However, there was no significant difference in the elongation factor between M1 and M2 macrophages when 10% hPL was used. In addition, the elongation factor of M2 macrophages cultured under 10% FBS conditions was significantly higher than that obtained by culturing with 5% hPL (M2: 10% FBS 12.84 ± 0.8333 versus 5% hPL 3.728 ± 0.9190, *p* < 0.05) (Figure 2C).

### 2.3. FBS or hPL Culture Supplementation Induces Different Phenotypes of M1 and M2 Macrophages

**Figure 3:**
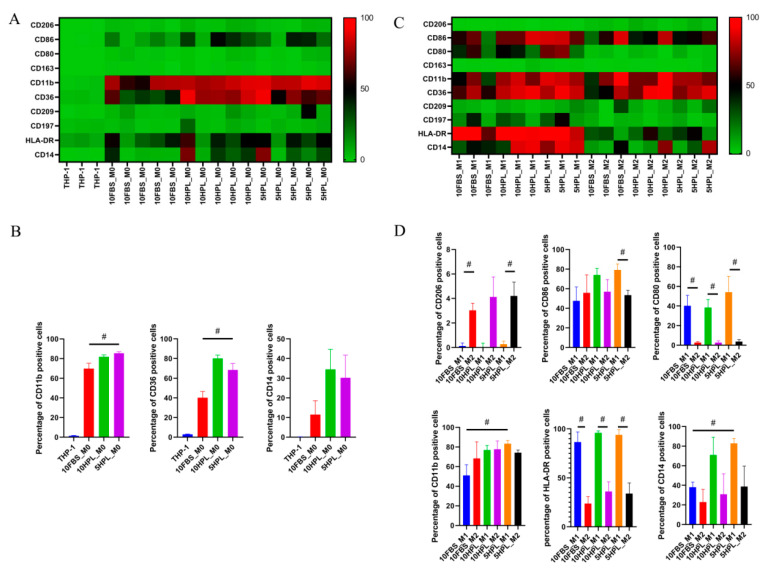
Analysis of surface marker expression of undifferentiated THP-1 cells and induced M0, M1, and M2 macrophages under FBS or hPL supplementation by flow cytometry. (**A**) Heat map of THP-1 cells and M0 macrophages’ surface marker expression. (**B**) Surface expression of CD11b, CD36, and CD14 in THP-1 and M0 macrophages (*n* = 3–5, # *p* < 0.05). (**C**) Heat map of M1 and M2 macrophage surface marker expression. (**D**) Surface expression of CD206, CD86, CD80, CD11b, HLA-DR, and CD14 in M1 and M2 macrophages (*n* = 3, # *p* < 0.05).

M0/M1/M2 macrophage-related cell surface markers, CD206, CD86, CD80, CD163, CD11b, CD36, CD209, CD197, HLA-DR, and CD14, were quantified by flow cytometry under 10% FBS, 10% hPL, or 5% hPL supplementation. The expression of the abovementioned surface molecules by M0 macrophages compared to untreated THP-1 cells are shown in a heat map in Figure 3A and illustrated as diagrams in Figure 3B. The results show that, compared with the M0 macrophages cultured in 10% FBS, the percentage of CD11b- and CD36-positive cells in M0 macrophages cultured in 5% hPL was significantly increased (CD11b M0: 10% FBS 69.94 ± 5.492 versus 5% hPL 85.43 ± 1.579, *p* < 0.05; CD36 M0: 10% FBS 40.16 ± 6.491 versus 5% hPL 68.53 ± 6.599, *p* < 0.05) (Figure 3B).

Surface marker expressions on M1 compared to M2 macrophages are illustrated in the heat map showed in Figure 3C and as diagrams in Figure 3D. Using different culture supplementation, the percentages of CD80- and HLA-DR-positive M1 macrophages were significantly increased compared to those of M2 macrophages (5% hPL CD80: M1 54.18 ± 16.11 versus M2 3.782 ± 1.893, *p* < 0.05; 5% hPL HLA-DR: M1 93.96 ± 5.226 versus M2 33.98 ±10.80, *p* < 0.05). Additionally, the results showed that, compared with M1 macrophages cultured under 10% FBS, the percentages of CD14- and CD11b-positive M1 macrophages cultured were significantly increased (CD14 M1: 10% FBS 38.11 ± 5.145 versus 5% hPL 82.90 ± 5.059, *p* < 0.05; CD11b M1: 10% FBS 51.03 ± 10.84 versus 5% hPL 83.29 ± 3.404, *p* < 0.05) under 5% hPL supplementation. The expression of CD86 reached no significant differences between M1 and M2 macrophages under 10% FBS and 10% hPL supplementation. However, CD86 percentages in M1 macrophages cultured under 5% hPL supplementation were significantly higher than those of M2 macrophages (5% hPL CD86: M1 79.13 ± 6.255 versus M2 53.52 ± 4.851, *p* < 0.05). Under 10% FBS and 5% hPL conditions, the percentages of CD206-positive M2 macrophages were significantly higher than those of M1 macrophages (5% hPL CD206: M1 0.2654 ± 0.2511 versus M2 4.213 ± 1.121, *p* < 0.05), but when using 10% hPL, there was no significant difference between M1 and M2 macrophages concerning the CD206-positive cells (Figure 3D).

### 2.4. Direct Coculture of JPCs and M1/M2 Macrophages

**Figure 4:**
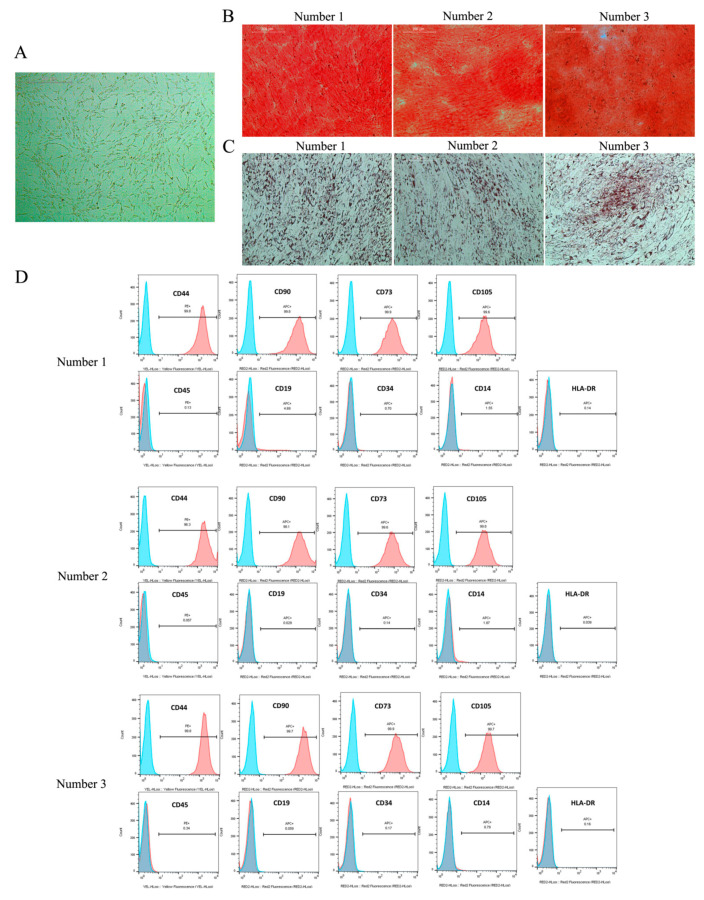
Phenotypic characterization of JPCs. (**A**) JPCs derived from 3 patients (number 1–3) demonstrate a fibroblastic-like appearance; scale bar: 500 μm. (**B**) Alizarin red staining shows that JPCs can undergo osteogenic differentiation (from three donors); scale bar: 200 μm. (**C**) Oil red O staining reveals adipogenic differentiation potential of JPCs (from three donors); scale bar: 200 μm. (**D**) JPCs show high expression of CD44, CD90, CD73, and CD105, but do not express CD45, CD19, CD34, CD14, and HLA-DR (from three donors).

The MSC phenotype of JPCs was demonstrated by a fibroblast-like morphology and adherent growth. Differentiation experiments showed that JPCs have osteogenic and adipogenic differentiation capability (Figure 4A–C). Additionally, expressions of CD44, CD90, CD73, and CD105 and the absence of hematopoietic stem cell markers were detected by flow cytometry in cells from 3 different patients (Figure 4D) [20].

**Figure 5:**
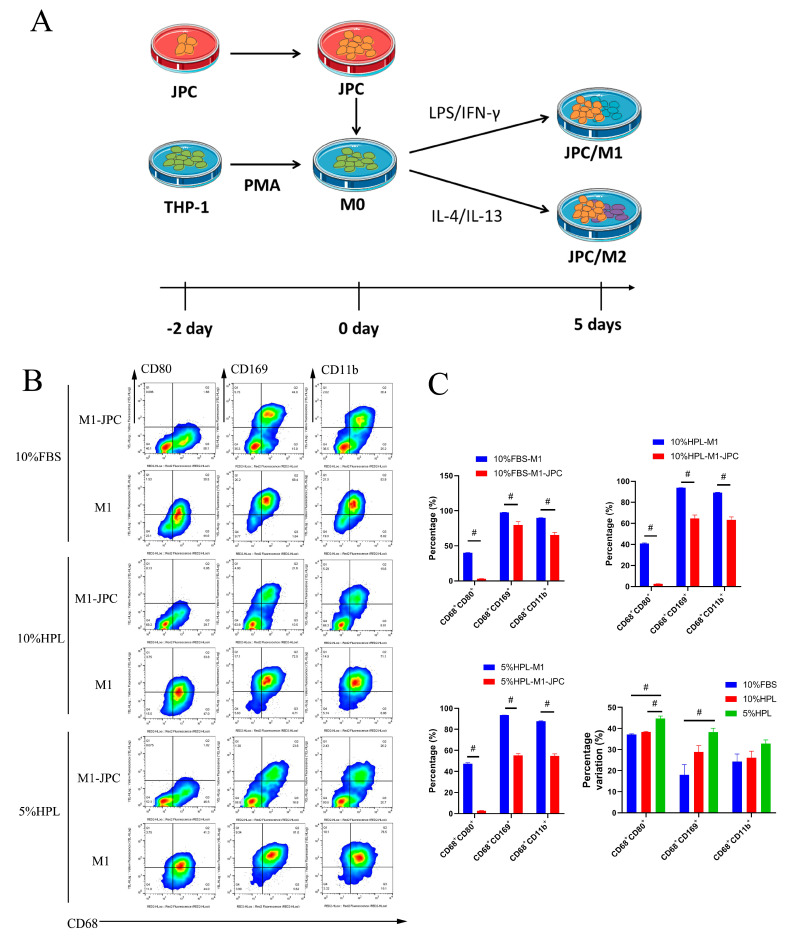
Surface marker expression by M1 macrophages after direct coculture with JPCs under FBS or hPL culture supplementation. (**A**) Schematic diagram of the experimental procedure for the contact coculture experiment of JPCs and macrophages. (**B**) Representative flow cytometric dot plots for CD80, CD169, and CD11b expressions in CD68^+^ macrophages in monocultures (M1) and cocultures (M1-JPC). (**C**) Percentages of CD80, CD169, and CD11b expressions and percentage variation in monocultures (M1) and cocultures (M1-JPC), depending on the used culture supplementations (*n* = 3, # *p* < 0.05).

PMA-induced THP-1 cells (M0) were cultured for two days under the different supplementations. At the same time, JPCs were cultured separately, also for two days under 5% hPL supplementation (Figure 5A). Thereafter, JPCs and M0 macrophages were cocultured for an additional five days under M1- and M2-inductive conditions (Figure 5A). For flow cytometric analysis of cell surface marker expression on M1 macrophages (Figure 5B,C), CD68^+^ cells were gated to identify macrophages in the detached cell monolayer. CD80 and CD11b expressions were used to determine the differentiation of M0 to M1 macrophages, and CD169^+^ cells were also detected (Figure 5B). Compared with the monoculture groups, the percentages of CD68^+^CD80^+^-, CD68^+^CD11b^+^-, and CD68^+^CD169^+^-positive cells were significantly decreased in all direct coculture groups. However, we found the highest differences in the percentages of CD68^+^CD80^+^ and CD68^+^CD169^+^ cells under 5% hPL conditions compared to those obtained under 10% FBS (CD68^+^CD80^+^: 5% hPL-M1 47.34 ± 0.9195 versus 5% hPL-M1-JPC 2.641 ± 0.3158, *p* < 0.05; CD68^+^CD169^+^: 5% hPL-M1 93.52 ± 0.05527 versus 5% hPL-M1-JPC 55.19 ± 1.759, *p* < 0.05). No significant differences in the percentages of variation of CD68^+^CD11b^+^ cells were detected between the three culture conditions (Figure 5C).

**Figure 6:**
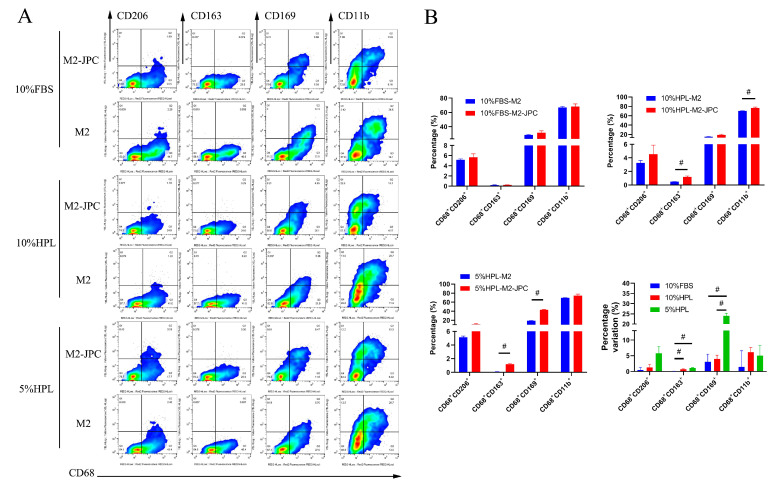
Surface marker expression by M2 macrophages after direct coculture with JPCs under FBS and hPL culture supplementation. (**A**) Representative flow cytometric dot plots for CD206, CD163, CD169, and CD11b protein expressions in macrophages in monocultures (M2) and cocultures (M2-JPC). (**B**) CD206, CD163, CD169, and CD11b protein expressions and percentage variation in monocultures (M2) and cocultures (M2-JPC), depending on the used culture supplementations (*n* = 3, # *p* < 0.05).

For flow cytometric analysis of cell surface marker expression on M2 macrophages (Figure 6), CD68^+^ cells were gated to identify macrophages in the mixed cell suspension and CD206, CD163, and CD11b were used to determine the differentiation to M2 macrophages. Further, CD169^+^ macrophages were also detected (Figure 6A). When using 10% FBS as a culture supplement, no significant differences were found between the coculture and monoculture groups for any of the abovementioned cell surface markers. When using 10% hPL as a culture supplement, the percentages of CD68^+^CD163^+^ and CD68^+^CD11b^+^ cells in the coculture group were significantly increased compared with those from the monoculture group (CD68^+^CD163^+^: 10% hPL-M2 0.4773 ± 0.04137 versus 10% hPL-M2-JPC 1.187 ± 0.1320, *p* < 0.05; CD68^+^CD11b^+^: 10% hPL-M2 70.22 ± 0.6602 versus 10% hPL-M2-JPC 76.35 ± 2.021, *p* < 0.05). When using 5% hPL as a culture supplement, the percentages of CD68^+^CD163^+^ and CD68^+^CD169^+^ cells in the coculture groups were increased compared with those of the monoculture groups (CD68^+^CD163^+^: 5% hPL-M2 0.08298 ± 0.02422 versus 5% hPL-M2-JPC 1.216 ± 0.1043, *p* < 0.05; CD68^+^CD169^+^: 5% hPL-M2 18.86 ± 0.7340 versus 5% hPL-M2-JPC 42.88 ±1.085, *p* < 0.05). Additionally, we found that the variation in the percentages of CD68^+^CD163^+^ and CD68^+^CD169^+^ cells cultured in 5% hPL were higher than that in 10% FBS (CD68^+^CD163^+^: 10% FBS 0.03800 ± 0.08057 versus 5% hPL 1.133 ± 0.1022, *p* < 0.05; CD68^+^CD169^+^: 10% FBS 3.079 ± 2.414 versus 5% hPL 24.02 ± 1.254, *p* < 0.05). There were no significant differences in the variation of the percentages of CD68^+^CD206^+^ and CD68^+^CD11b^+^ cells between the three culture supplementation groups (Figure 6B).

### 2.5. Fluorescent Cell Tracking of JPCs/M1/M2 Macrophages in Direct Cocultures

**Figure 7:**
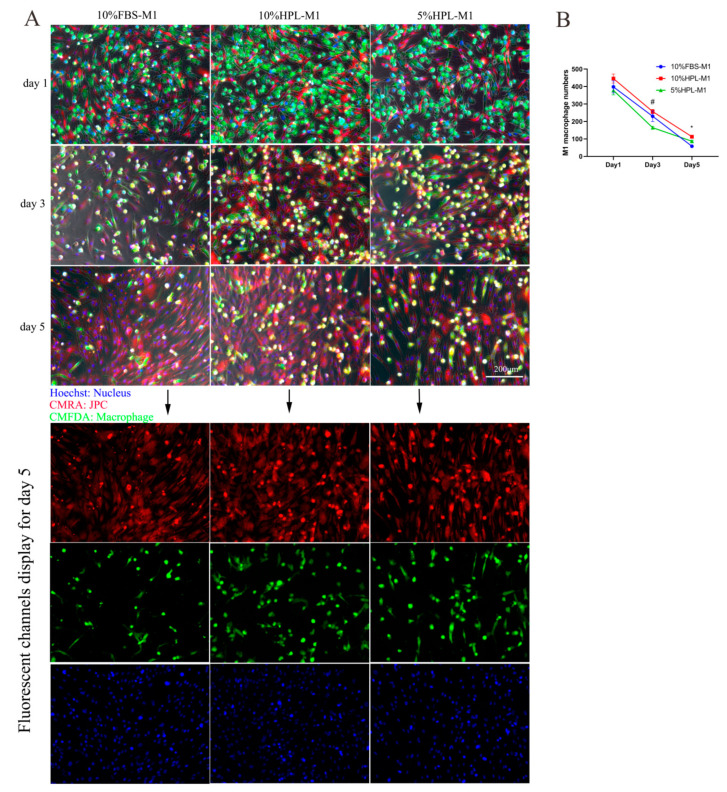
Cell tracking of JPC and M1 macrophages cultured under 10% FBS, 10% hPL, or 5% hPL culture supplementation by fluorescence microscopic imaging. (**A**) Representative fluorescence micrographs of JPCs and M1 macrophages on the first, third, and fifth days of coculture (blue: nucleus; red: JPC; green: macrophage); scale bar: 200 μm. (**B**) M1 macrophage numbers counted in the fluorescence images at days 1, 3, and 5 of coculture with JPCs (*n* = 3, 5% hPL compared with 10% FBS * *p* < 0.05, 5% hPL compared with 10% hPL # *p* < 0.05).

**Figure 8:**
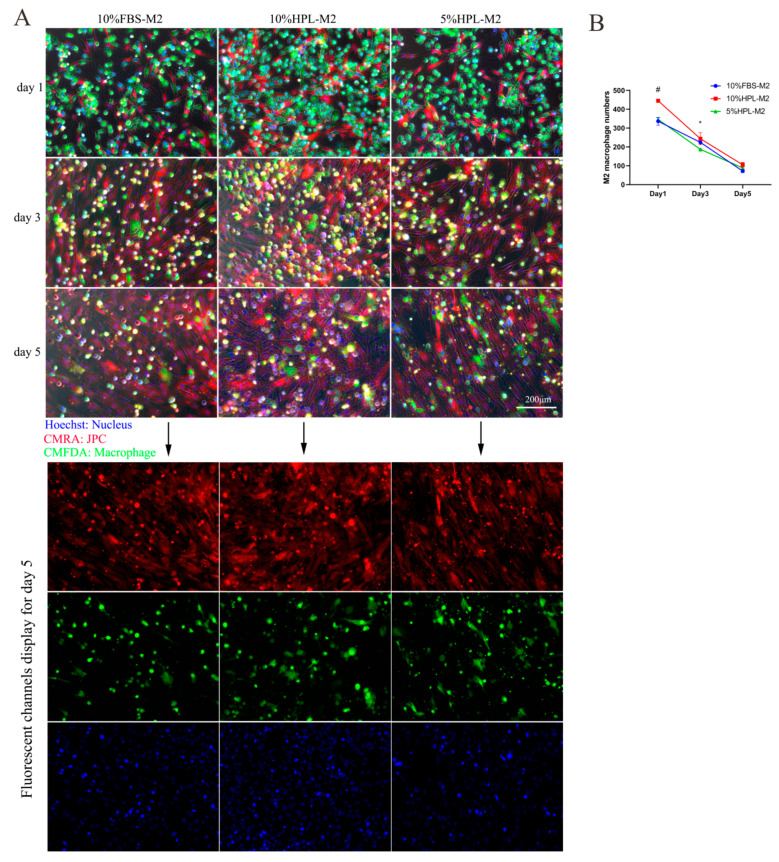
Cell tracking of JPCs and M2 macrophages cultured under 10% FBS, 10% hPL, or 5% hPL supplementation by fluorescence microscopic images. (**A**) Representative fluorescence micrographs of JPCs and M2 macrophages on the first, third, and fifth days of coculture (blue: nucleus; red: JPC; green: macrophage); scale bar: 200 μm. (**B**) M2 macrophage numbers counted in the fluorescence images at days 1, 3, and 5 of coculture with JPCs (*n* = 3, 5% hPL compared with 10% FBS * *p* < 0.05, 5% hPL compared with 10% hPL # *p* < 0.05).

The results of cell tracking experiments showed that macrophages labeled with green CMFDA and JPCs labeled with red CMRA can be distinguished in the direct coculture system. On the first day of direct coculture, JPCs were in an initial adherent state, marked as bright red, and macrophages were marked as bright green. On the third and fifth days of cell coculture, JPCs were still red, while some of the marked macrophages gradually turned to yellow (red mixed with green), which was probably based on the fact that green macrophages internalized substances from the red-labeled JPC (Figure 7A and Figure 8A).

By ImageJ analysis, M1 macrophage numbers were counted in the fluorescence microscopic images. The results showed no significant differences between the three types of culture supplementation at day 1. However, at day 3, M1 macrophage numbers were shown to be significantly lower under 5% hPL supplementation compared with 10% hPL supplementation. Additionally, at day 5, the number of M1 macrophages was significantly reduced under 5% hPL compared to 10% FBS culture conditions (Figure 7B).

Concerning the cell number of M2 macrophages, they were significantly lower in 5% hPL at day 1 compared with 10% hPL culture conditions. At day 3, M2 numbers in 5% hPL were shown to be significantly lower compared with 10% FBS culture conditions. However, no significant differences between the three types of culture supplementation were detected at day 5 (Figure 8B).

### 2.6. Typical M1/M2 Macrophage Morphology in the Coculture System under 5% hPL Supplementation

On the first day of JPC/macrophage cocultures in 5% hPL, both macrophages and JPCs were attached to the plastic support and the macrophages showed pseudopodia interacting apparently with the JPCs (Figures S1 and S2).

At day 5 of coculture in 5% hPL, JPCs covered the surface of the culture plate. Most macrophages were apparently attached to the surface of the JPC monolayer and turned yellow. In the JPC/M1 coculture system, the typical macrophage showed a spindle-shaped cell body, a round nucleus, one long pseudopod, and a bifurcation at the end of the pseudopod. Additionally, the pseudopodia of M1 macrophages were apparently in close contact with JPCs, and the ends of pseudopodia were attached to the JPCs’ membranes (Figure S1).

In the JPC/M2 coculture system, the typical macrophage cell body displayed a rather round shape, the nucleus was also round, and cells possessed a higher number of pseudopodia, which were shown to be more ramified than those of M1 cells. Additionally, M2 macrophages apparently interacted with JPCs. Furthermore, some stained particles between the cells, which were visible at day 1, increased in number at day 5. Some red particles were visible within the cytoplasm of M2 macrophages (Figure S2).

## 3. Discussion

To our knowledge, there are no other reports focusing on the comparison of macrophage polarization derived from THP-1 cells under FBS and hPL culture supplementation. Pursuing this goal, we compared cell morphology and surface marker expression of M1 and M2 macrophages differentiated under the different culture conditions.

Macrophages derived and differentiated from the monocytic cell line THP-1 have been widely used for macrophage polarization studies [21]. In a first step, THP-1 cells can be differentiated into immature M0 macrophages by using PMA, and high PMA concentrations were used to obtain pro-inflammatory M1 macrophages [22]. Our experience has shown that PMA concentration and incubation time are crucial in order to obtain distinct macrophage phenotypes. Therefore, we applied different PMA concentrations (5 nM, 25 nM, and 150 nM) to stimulate THP-1 cells to immature macrophages (M0) for 48 h, and then LPS and IFN-γ or IL-4 and IL-13 were used for a further 24 or 72 h to stimulate M0 polarization into M1- or M2-like macrophages for a further 24 or 72 h. Results obtained from both flow cytometry and immunofluorescence staining showed that the 5 nM PMA concentration for M0 differentiation, followed by M1/M2 differentiation for further 72 h, could effectively promote differential surface marker expression of M1 and M2 macrophages. The cell elongation factor measurements of M1 and M2 macrophages showed opposite patterns when the cells were differentiated under 10% FBS, compared to 5% hPL supplementation. Under 5% hPL conditions, the pseudopodia of M1 macrophages were shown to be longer than those of M2 macrophages, while the opposite was observed under 10% FBS supplementation. Thus, cell morphology obtained under 5% hPL culture conditions was more consistent with the expected phenotype, due to the fact that, usually, M1-type macrophages have a flat morphology with spindle-shaped pseudopodia [23].

The surface markers CD14 and CD11b represent common surface markers for macrophages, whereas CD80 and CD86 are considered to be specific markers for M1 macrophages [24,25,26]. In our experiments, 5% hPL supplementation was beneficial for the CD14 and CD11b expression in immature M0 macrophages. Further, CD86 expression of M1 macrophages cultured in 5% hPL was significantly higher compared to CD86 expression in M2 macrophages, whereas no significant differences between M1 and M2 macrophages were detected in 10% FBS- and 10% hPL-supplemented medium. Similarly, M1 macrophages cultured in 5% hPL expressed higher levels of CD14 and CD11b compared to 10% FBS supplementation. Additionally, significantly different levels of CD80 and HLA-DR expression between M1 and M2 macrophages were detected under 10% hPL or 5% hPL culture conditions.

Our results indicated a more distinct polarization of macrophages achieved with low-concentrated hPL compared to FBS supplementation and led us to the conclusion that hPL is the better choice for M1 and M2 polarization studies using THP-1 cells.

MSCs derived from jaw periosteal tissue provide an ideal stem cell source for bone tissue engineering applications in maxillofacial surgery. In the present study, both surface marker expression and multilineage differentiation capability showed that JPCs meet the definition of MSCs held by the international society for cellular therapy [27]. Further, our previous research results of quantitative analysis of the mineralization potential of JPCs and examinations by nanoindentation and Raman spectroscopy technology demonstrated that hPL supplementation can effectively promote the proliferation activity and mineralization ability of jaw periosteal cells compared with FBS culture conditions [17,28,29].

MSCs are capable to regulate and suppress immune responses, making them attractive not only in the field of regenerative medicine, but also for the treatment of autoimmune diseases and organ transplantations. In previous studies, we investigated the immunomodulatory functions of JPCs for the first time by clarifying their effects on dendritic cell maturation [5,6]. In the present study, we analyzed for the first time the interactions between JPCs and macrophages under different culture supplementations. The recent study from Tylek and co-authors could demonstrate that hPL outperforms FBS in coculture experiments of femoral head-derived MSCs and macrophages [30]. However, in the abovementioned work, flow cytometry analysis could not be performed due to low cell numbers collected after FBS supplementation. Further, dexamethasone was used to induce and activate M2 macrophages in the coculture of femoral head-derived MSCs and M2 macrophages. Other studies confirmed that the glucocorticoid dexamethasone can promote the differentiation of macrophages into M2-type macrophages [31] and induce macrophage apoptosis [32,33,34]. Interestingly, dexamethasone can strongly influence the anti-inflammatory and immunomodulatory effects of MSCs [35]. Therefore, the use of dexamethasone in coculture studies of macrophages and MSCs still needs further verification. In our coculture experiments, we first used THP-1 monocytes to differentiate into immature M0 macrophages and then analyzed the effect of JPCs on the polarization of M1 or M2 macrophages in a pro-inflammatory (LPS and IFN-γ) or anti-inflammatory (IL-4 and IL-13) microenvironment. This coculture model contained no interfering hormones, allowing a more accurate observation of the effects of JPCs on macrophage polarization.

Macrophages are highly heterogeneous cells that rapidly change their phenotype in response to microenvironmental signals [36]. New research shows that in addition to M1 and M2 macrophages, there are also CD169^+^ macrophages, which do not mediate phagocytosis but are mainly involved in immune regulation rather than in maintaining homeostasis [9,37]. Our results from the coculture experiments demonstrated that JPCs inhibited the polarization of immature M0 to M1-type macrophages, while reducing the percentage of CD169^+^ macrophages. However, JPCs cultured in 5% hPL could more effectively reduce the numbers of CD68^+^CD80^+^ (M1) and CD68^+^CD169^+^ macrophages, compared to 10% FBS culture conditions. Additionally, JPCs supplemented with 5% hPL could also increase the numbers of CD68^+^CD163^+^ as well as CD68^+^ CD169^+^ macrophages during M2 polarization. These results showed that JPCs’ ability to inhibit the polarization of M0 to M1 macrophages, as well as to promote the M2 phenotype and to regulate CD169^+^ macrophages, can be enhanced by a low concentration of hPL.

By the cell tracking approach, we could visualize the two cell types in the direct coculture system. After calculation of macrophage numbers, we detected significantly lower numbers of M1 macrophages under 5% hPL supplementation on the third and fifth days of cocultivation with JPCs. In addition, JPCs/M2 cocultures showed also decreased M2 numbers at day 1 and day 3 under 5% hPL compared with other supplementations. However, at day 5, M2 numbers seemed to be equal regardless of different culture supplementation. These findings indicate that numbers of M1/M2 macrophages can be decreased in coculture with JPCs under 5% hPL conditions compared to other supplementations. The underlying mechanisms for the observed effects of JPCs in combination with culture supplementation needs to be further investigated. Besides, one point about using hPL to simulate human physiological host immune environment also needs more investigations. The THP-1 macrophage polarization, regulated by JPCs by using hPL supplement in our study, was limited to phenotypic research of macrophages polarization in vitro. More studies about physiological relevance in using hPL instead of FBS need to be verified by individual samples and in vivo studies.

The classical function of macrophages is to engulf foreign substances. Macrophages activated by inflammatory factors can extend pseudopodia, increase the numbers of lysosomes, and enhance phagocytotic and secretion activities [38]. The morphology and length of macrophage pseudopodia reflect macrophages’ ability to migrate and capture foreign substances [39]. Studies have shown that using a micropatterning approach to generate long-shaped macrophages under FBS culture supplementation led to the typical cytokine release pattern of the anti-inflammatory (M2) phenotype [40]. This finding is consistent with the result of our study where M2 macrophages under FBS culture supplementation were more elongated. However, we demonstrated that emerged pseudopodia of M1 or M2 macrophages exhibited completely opposite patterns in FBS medium compared to hPL condition. Under 5% hPL supplementation, which seemed to be beneficial to generate distinct M1 and M2 phenotypes, we detected M1-type macrophages with one long pseudopod, while M2-type macrophages showed shorter pseudopodia and larger cell bodies on the fifth day of coculture. Additionally, macrophages attached to the surface of JPCs through pseudopodia. Even though JPCs were seeded onto the layer of macrophages, they did not cover them, indicating that macrophages are able to move around in order to conduct phagocytosis. Interestingly, we also found that the CMFDA-labeled macrophages showed green fluorescence on the first day of cocultivation, but, on the third and fifth day, macrophages turned from green to yellow (yellow color is presented though red mixed with green color). However, the CMRA-labeled JPCs remained still red on the fifth day, indicating that the macrophages internalized substances released by JPCs and changed from green to yellow.

## 4. Materials and Methods

### 4.1. Cell Culture of the Monocytic Cell Line THP-1

Monocytic THP-1 cell lines were purchased from the American Type Culture Collection (ATCC, Gaithersburg, MD, USA) and expanded in RPMI 1640 medium (Thermo Fisher Scientific, Waltham, USA) containing 10% heat-inactivated FBS (Sigma-Aldrich, Darmstadt, Germany), 1% penicillin/streptomycin (Lonza, Basel, Switzerland), 1% Amphotericin B (Biochrom AG, Berlin, Germany), and 0.05 nM 2-mercaptoethanol (Sigma-Aldrich, Darmstadt, Germany). Cells were cultured in 75-cm^2^ flasks at a 37 °C in a 5% CO_2_-humidified incubator and medium was changed every other day. For passaging, THP-1 cells were centrifuged at 1400 rpm for 5 min at 8 °C and split in a ratio of 5 × 10^5^ to new T75 flasks.

### 4.2. Cell Culture and Identification of JPCs

According to the regulations of the local ethics committee (No. 618/2017BO2), JPCs from three donors (age: 20–31 years old; two males and one female) were included in this study after obtaining written, informed consent. JPCs were passaged and expanded in DMEM/F12 (Thermo Fisher Scientific, Waltham, MA, USA) containing 5% hPL, and cells of the fourth passage were used in all coculture experiments. The mesenchymal phenotypes (CD44, CD90, CD73, and CD105) of JPCs were characterized by flow cytometry, and the stem cell potential of JPCs was confirmed by osteogenic and adipogenic differentiation. In brief, JPCs were treated with osteogenic medium (100 μM vitamin C, 4 μM dexamethasone. and 10 mM β-glycerophosphate) or adipogenic medium (1 μM dexamethasone, 0.2 mM indomethacin, 10 μg/mL insulin, and 0.5 mM 3-isobutyl-1-methylxanthin) for 14 days. After cell fixation, osteogenic and adipogenic staining was performed through alizarin red or oil red O, respectively (Figure 4).

### 4.3. Establishment of an Optimal Protocol for Macrophage Polarization

Phorbol-12-myristate-13-acetate (PMA, Sigma-Aldrich, Darmstadt, Germany) was used in different concentrations to induce THP-1 cell differentiation to M0 macrophages. Therefore, 6 × 10^5^ THP-1 cells of passage 16 were resuspended in 10% FBS RPMI 1640 medium containing 5 nM, 25 nM, or 150 nM PMA, seeded per well of a six-well plate and incubated for 48 h. For further polarization, 15 ng/mL lipopolysaccharide (LPS, Sigma-Aldrich, Darmstadt, Germany) and 20 ng/mL interferon-γ (IFN-γ, Sigma-Aldrich, Darmstadt, Germany) were added to the culture to induce M1 generation, and 20 ng/mL interleukin 13 (IL-13, Sigma-Aldrich, Germany) and 20 ng/mL interleukin 4 (IL-4, Sigma-Aldrich, Darmstadt, Germany) to induce M2 polarization. Cells were stimulated to M1/M2 macrophages for 24 and 72 h. Flow cytometry and immunofluorescence staining were used to assess macrophage-specific protein expression (CD80 for M1 and CD206 for M2) on the cell surface.

### 4.4. Immunofluorescence Staining

M1 and M2 macrophages were washed with PBS three times and fixed with 4% paraformaldehyde for 30 min. Cells were washed with PBS three times and incubated with 10% goat serum (Abcam, Cambridge, UK) for 1 h to block non-specific protein–protein binding. Then, cells were incubated with the rabbit monoclonal anti-CD80 antibody (1:500 dilution)/mouse monoclonal anti-CD68 (1:100 dilution) antibody or rabbit monoclonal anti-CD206 (1:200 dilution)/mouse monoclonal anti-CD68 (1:100 dilution) (Abcam, Cambridge, UK) antibody overnight at 4 °C. Then, the cells were incubated with goat secondary anti-mouse IgG Alexa Fluor^®^ 568 antibody (1:500 dilution) and goat polyclonal secondary anti-rabbit IgG Alexa Fluor^®^ 488 antibody (1:500 dilution) (Abcam, Cambridge, UK). Nuclei were counterstained with Hoechst 33342 (1 μg/mL, Promocell, Heidelberg, Germany) for 5 min. After washing with PBS, macrophages were visualized on an Observer Z1 fluorescence microscope (Zeiss, Oberkochen, Germany). Image J software was used to quantify the total number of cells by counting nuclei (blue fluorescence), CD206^+^ cells (green fluorescence), and CD68^+^ cells (red fluorescence).

### 4.5. Comparison of Macrophages’ Polarization under FBS and hPL Supplementation

M1/M2 macrophage polarization was performed and compared under different conditions: RPMI 1640 medium containing 10% FBS, 10% hPL, or 5% hPL. The hPL supplement was provided by the Institute for Clinical and Experimental Transfusion Medicine of the University Hospital Tübingen. The hPL was fibrinogen-depleted. It did not contain heparin and was referred to as a research lysate based on the absent quarantine period. Using the three culture supplementations, 6 × 10^5^ THP-1 cells of passage 16 were seeded for M0 macrophage differentiation by using 5 nM PMA for 48 h. Further induction to M1 or M2 macrophages by the addition of 15 ng/mL LPS and 20 ng/mL IFN-γ (M1 polarization medium) or 20 ng/mL IL-13 and 20 ng/mL IL-4 (M2 polarization medium) followed for 72 h. M0, M1, and M2 macrophages cultured under FBS or hPL conditions were analyzed with an inverted light microscope (Leica Microsystems, Wetzlar, Germany) and photographed. ImageJ software was used to measure the cell area and the elongation factors (length of the long axis divided by length of the short axis) of M0, M1, and M2 macrophages. Flow cytometry was used to analyze the expression of the macrophage surface markers CD206 (PE, clone 15-2), CD86 (PE, clone BU63), CD80 (PE, clone 2D10), CD163 (PE, clone GHI/61), CD11b (PE, clone ICRF44), CD36 (APC, clone 5-271), CD209 (APC, clone 9E9A8), CD197 (APC, clone G043H7), HLA-DR (APC, clone L243), and CD14 (APC, clone M5E2) (Biolegend, San Diego, CA, USA).

### 4.6. Contact Coculture of JPCs and THP-1 Macrophages

The contact coculture experiment with THP-1 macrophages and JPCs from three donors was performed in a 24-well plate. The coculture experiments were divided into the 10% FBS, 10% hPL, or 5% hPL culture supplement groups, and each group was subdivided into a monoculture control group and a direct coculture experiment group.

Before coculture, JPCs (2 × 10^4^/well) were expanded for 48 h in 5% hPL DMEM/F12 medium in 24-well plates. Meanwhile, THP-1 cells (4 × 10^5^/well) seeded in other separate 24-well plates containing 10% FBS, 10% hPL, or 5% hPL RPMI1640 medium with 5 nM PMA were differentiated into M0 macrophages for 48 h.

For the direct coculture, JPCs were detached with TrypLE-Express (Thermo Fisher Scientific, Waltham, MA, USA) from the 24-well plate and resuspended in 10% FBS, 10% hPL, or 5% hPL RPMI 1640 medium containing LPS (15 ng/mL) and IFN-γ (20 ng/mL) or IL-13 (20 ng/mL) and IL-4 (20 ng/mL). Then, the JPC suspension was seeded into the corresponding wells containing FBS- or hPL-cultured M0 macrophages (refer to Figure 5A for the illustration).

For the monoculture group, cell-free 10% FBS, 10% hPL, or 5% hPL RPMI 1640 medium containing LPS and IFN-γ or IL-13 and IL-4 was added to the macrophage monocultured wells.

After five days of contact coculture with JPCs and M1-stimulated macrophages or JPCs and M2-stimulated macrophages, phenotypic analysis of macrophages was performed by flow cytometry to measure the macrophage polarization state. CD68 was used to distinguish between macrophages in the direct coculture system and monoculture system. CD80 (M1 marker) or CD206 and CD163 (M2 markers) were used to determine the polarization state of M1 or M2 macrophages. CD169 was used to detect CD169^+^ macrophages.

### 4.7. Cell Tracking for the Detection of JPCs or THP-1 Macrophages in the Direct Coculture System

In order to distinguish between JPCs and macrophages in the coculture system, we used CellTracker^TM^ nontransferable fluorescent dyes (Thermo Fisher Scientific, Waltham, USA). On day 0, JPCs were labeled with red CMRA (20 μM), and M0 macrophages with green CMFDA (20 μM). After 30 min of incubation, the CellTracker solution was removed and the CMRA-labeled JPCs were detached and resuspended in 10% FBS, 10% hPL, or 5% hPL RPMI 1640 medium containing LPS and IFN-γ or IL-13 and IL-4. Then, CMRA-labeled JPCs’ suspension was seeded onto the corresponding supplementation of CMFDA-labeled M0 macrophages and cocultured for an additional five days.

Fluorescence images of JPCs and macrophages were taken on the first, third, and fifth days of coculture, and ImageJ software was used to quantify the cell number of macrophages. Nuclei were stained with Hoechst 33342 (1 μg/mL, PromoCell, Heidelberg, Germany) for 5 min and fluorescence microscopy was used for morphological analysis of JPCs and macrophages.

### 4.8. Flow Cytometric Measurements

After the medium was removed, coculture cell layers were washed with PBS. Cells were detached from the plate using TrypLE Express. After blocking the unspecific binding sites with 100 μL 10% Gamunex (human immune globulin solution) for 10 min on ice, cells were incubated with the required antibodies (all antibodies were purchased from the Biolegend company) for further 30 min on dark. After centrifugation, cells were washed twice with FACS buffer (PBS containing 0.1% BSA) and measured by the Guava EasyCyte 6HT-2L flow cytometer (Merck Millipore, Germany) immediately. FlowJo software (FlowJo LLC, Ashland, OR, USA) was used for data evaluation.

### 4.9. Statistical Analysis

The statistical values for all measurements are expressed as means ± standard error of means (SEM). All statistical analyses were carried out using the GraphPad Prism software (La Jolla, CA, USA). The two-tailed Student’s t-test was used to analyze significant differences. A *p* value < 0.05 was considered significant.

## 5. Conclusions

Compared with FBS and 10% hPL, culture supplementation with 5% hPL is superior for the generation of distinct THP-1-derived M1 and M2 macrophage phenotypes. Under hPL culture condition, JPCs were best able to effectively inhibit M1 polarization of macrophages in our direct coculture system. In addition, fluorescent dyes’ tracking method is a useful imaging technique for distinguishing JPC and macrophages in the direct coculture system.

## Data Availability

The data that support the findings of this study are available from the corresponding author upon reasonable request.

## Supplementary Materials

The following are available online at https://www.mdpi.com/article/10.3390/ijms22094310/s1. Figure S1: Typical macrophage morphology under 5% hPL culture supplementation on the first and fifth days of cocultivation of fluorochrome-labeled JPC and M1. Figure S2: Typical macrophage morphology under 5% hPL culture supplementation on the first and fifth days of cocultivation of fluorochrome-labeled JPC and M2.
