# Supplementary material for: Jaw Periosteum-Derived Mesenchymal Stem Cells Regulate THP-1-Derived Macrophage Polarization
Source: Int J Mol Sci. 2021 Apr 21;22(9):4310. doi: 10.3390/ijms22094310 (PMC8122347; doi:10.3390/ijms22094310)
Supplement: Supplementary file 1 [file ijms-22-04310-s001.zip › ijms-1190384-supplementary.pdf]

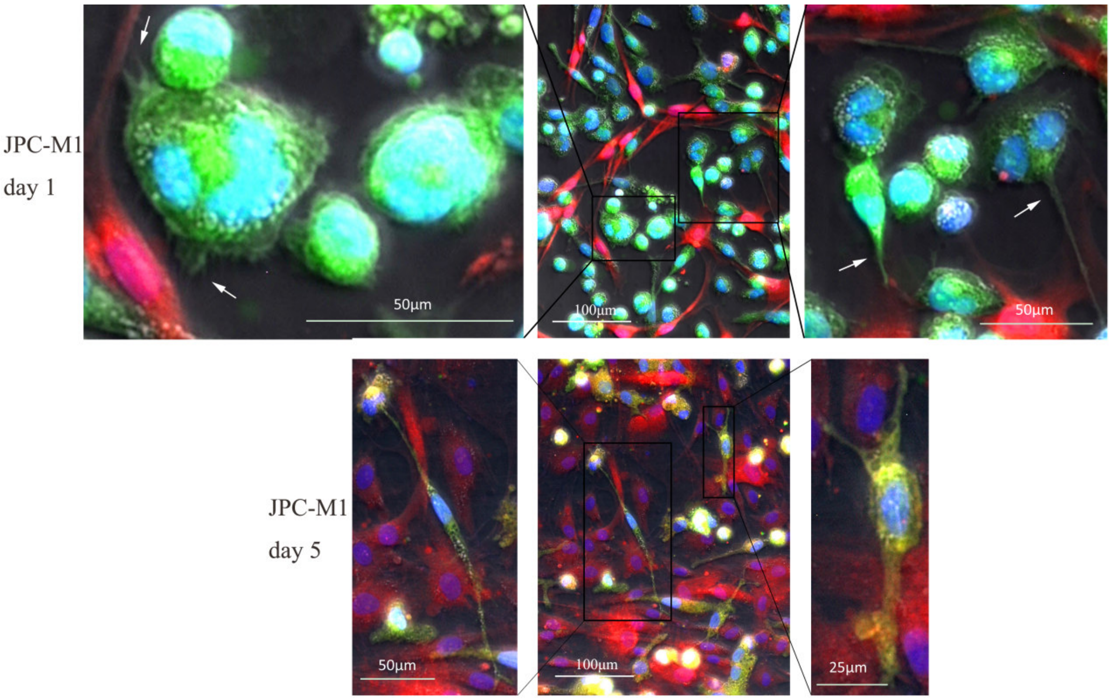

Figure S1. Analysis of fluorescently labeled M1 macrophages and JPCs morphology under 5% hPL culture supplementation on the first and fifth day of cocultivation. In the middle panel, overview images are shown and in the left and right panel magnified images are illustrated (White arrow: At the initial stage of coculture, pseudopods protrude from the side of macrophages in contact with JPCs).

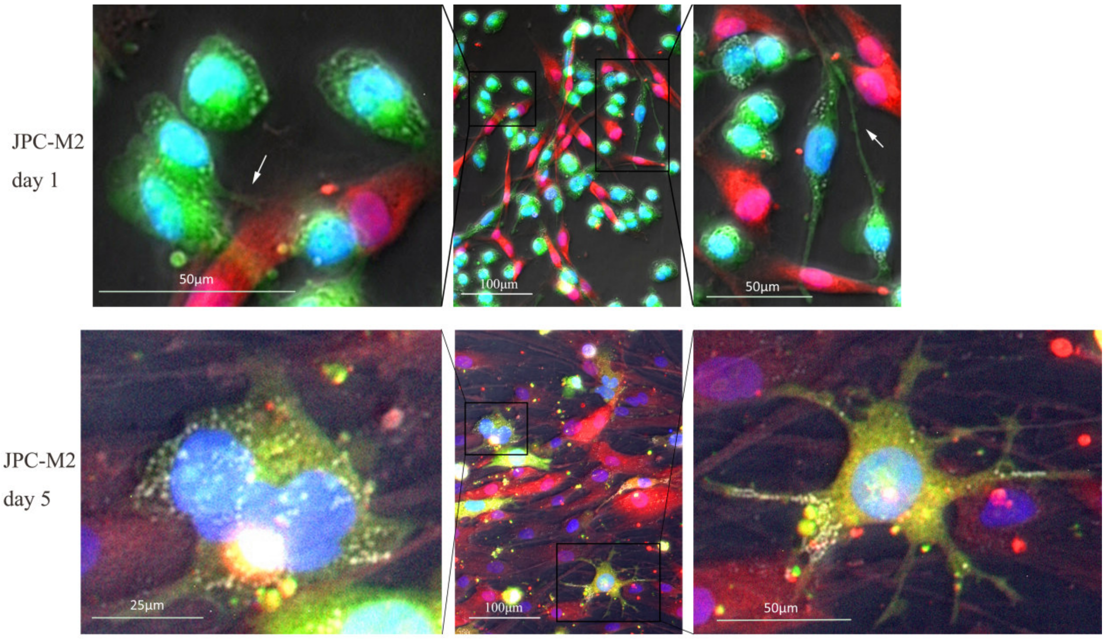

Figure S2. Analysis of fluorescently labeled M2 macrophages and JPCs morphology under 5% hPL culture supplementation on the first and fifth day of cocultivation. In the middle panel, overview images are shown and in the left and right panel magnified images are illustrated (White arrow: At the initial stage of coculture, pseudopods protrude from the side of macrophages in contact with JPCs).
